# Supplementary material for: Automatic rating of incomplete hippocampal inversions evaluated across multiple cohorts
Source: ArXiv. 2025 Jan 20:arXiv:2408.02496v2. Originally published 2024 Aug 5. Preprint. [Version 2] (PMC11326423)
Supplement: Supplement 1 [file NIHPP2408.02496V2-supplement-1.pdf]

Appendix A. Automatic rating methods of incomplete hippocampal inversions on various cohorts: supplementary material

A.1 Supplementary Figures

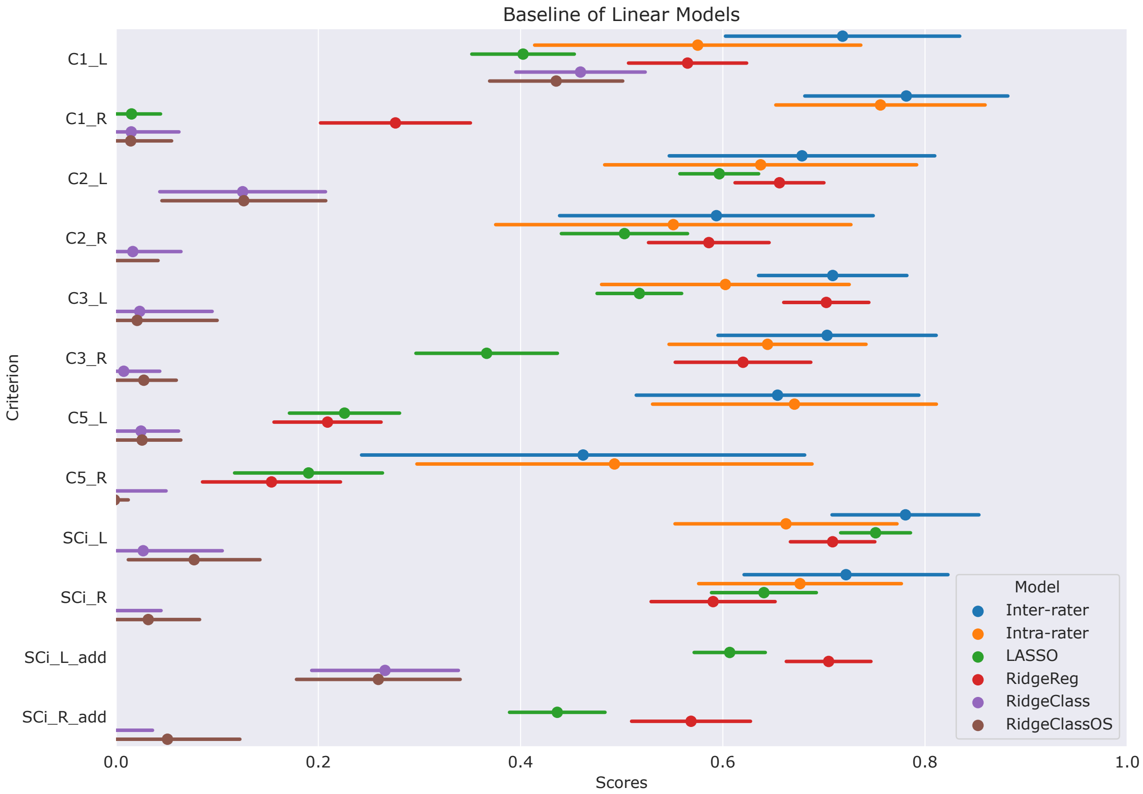

Figure A1: *Results obtained with additional linear models.* LASSO regression, ridge logistic regression classifier (denoted as RidgeClass), ridge logistic regression classifier with oversampling of the minority class (denoted as RidgeClassOS) were studied in addition to the ridge regression (denoted as Ridge) which is presented in the main manuscript. The figure displays the results of the predictions of individual criteria and composite scores on an independent test set of the IMAGEN database. We show the mean metrics (weighted Cohen's Kappa score for C1, C2 and C3, unweighted Cohen's Kappa score for C5 and ICC for composite scores) and 95% confidence intervals obtained through bootstraping.

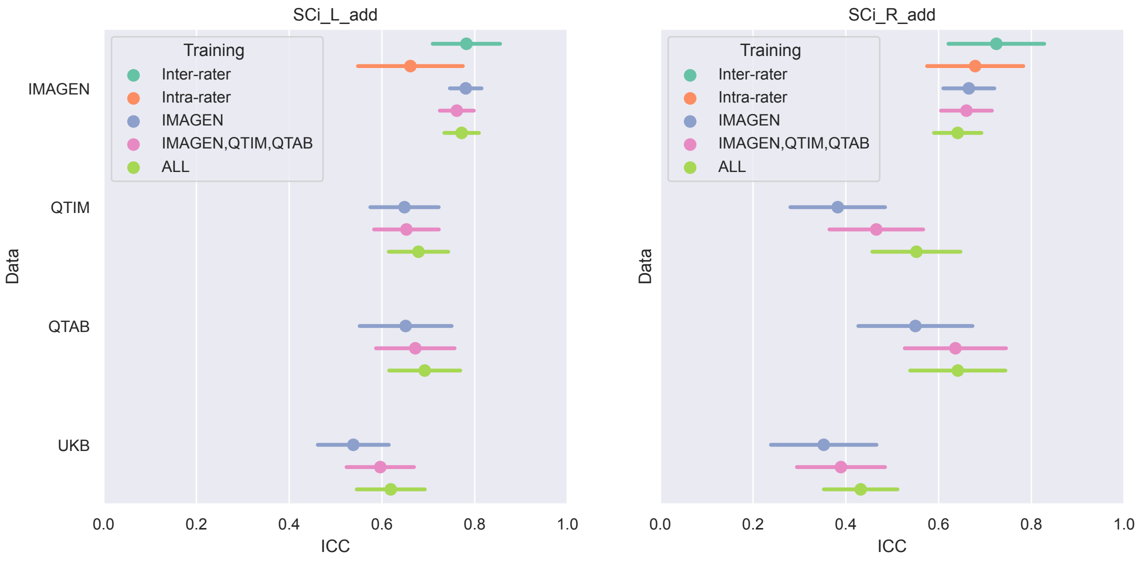

Figure A2: *Results of the predictions of composite scores shown separately on independent test sets of IMAGEN, QTIM, QTAB and UKBiobank.* We show the mean ICC and 95% confidence intervals obtained through bootstraping. Results are shown for the Conv5-FC3. Three training methods are compared: using only the training set of the IMAGEN database, using the training sets of IMAGEN, QTIM and QTAB databases and using the training sets of all databases (IMAGEN, QTIM, QTAB, UKBiobank).

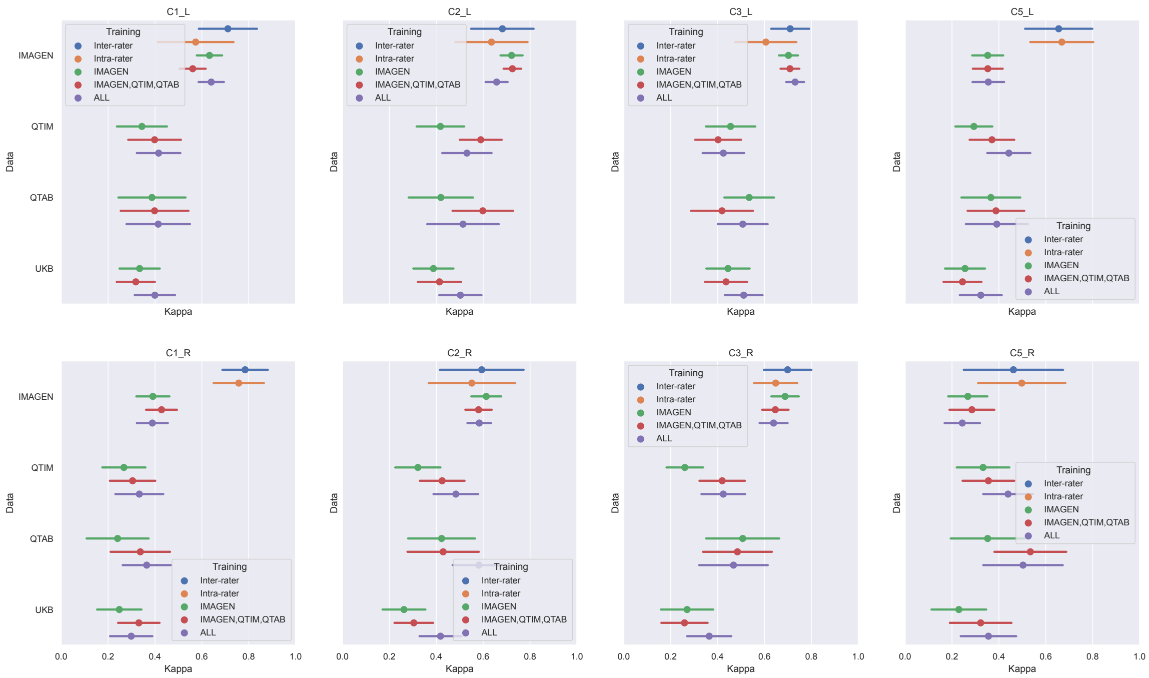

Figure A3: *Results of the predictions of individual criteria shown separately on independent test sets of IMAGEN, QTIM, QTAB and UKBiobank.* We show the mean metrics (weighted Cohen's Kappa score for C1, C2 and C3, unweighted Cohen's Kappa score for C5) and 95% confidence intervals obtained through bootstrapping. Results are shown for the Conv5-FC3. Three training methods are compared: using only the training set of the IMAGEN database, using the training sets of IMAGEN, QTIM and QTAB databases and using the training sets of all databases (IMAGEN, QTIM, QTAB, UKBiobank).

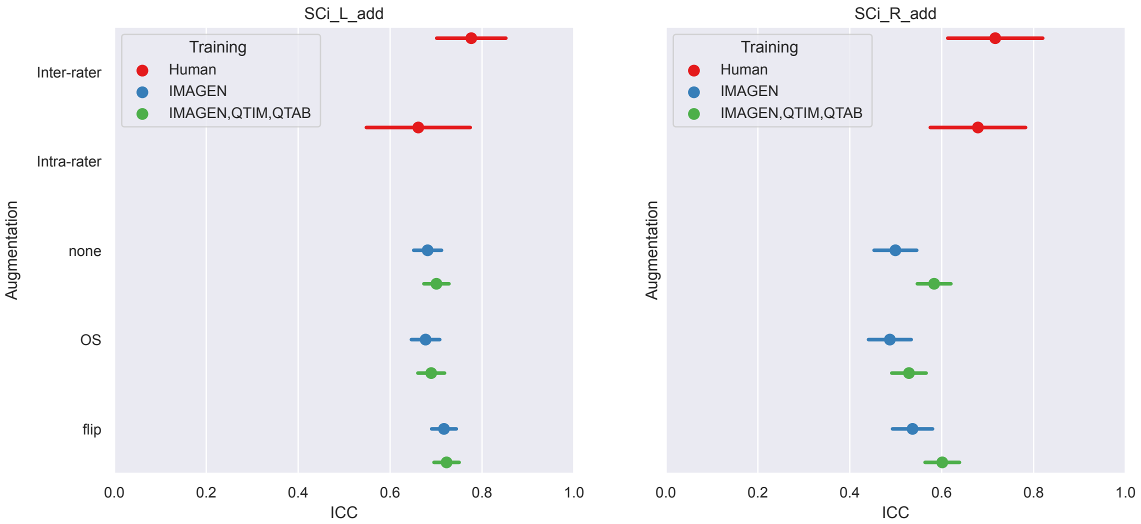

Figure A4: *Results of the predictions of composite scores on pooled independent test sets of the IMAGEN, QTIM, QTAB and UKB cohorts.* We show the mean ICC and 95% confidence intervals obtained through bootstrapping. Results are shown for the Conv5-FC3 model trained using only IMAGEN (single) and IMAGEN, QTIM and QTAB (multi), alongside inter-rater and intra-rater performances. Three data augmentation strategies are compared (none, over-sampling (OS), flip). These results are shown for predictions in the left and right hemispheres.

A.2 Supplementary Methods and Results

Methods

ROI selection: Three ROIs were tested on a ridge regression and a Conv5-FC3 net in a previous study and evaluated on the independent test-set of the IMAGEN cohort. ROIs were as follows:

- Hippocampus and close sulci: [24:96,54:107,16:49] in MNI coordinates
- Hippocampus and all surrounding sulci: [10:110,54:107,6:49] in MNI coordinates
- Temporal lobe: [10:110,15:107,6:79] in MNI coordinates

Data augmentation: As data, particularly in the right hemisphere, remains very unbalanced, we applied two data augmentation methods to improve our training. On one hand we simply over-sampled minority classes of each criterion by presenting the model with the same number of images from each class. This means that some images were shown repeatedly. Which images were shown several times was decided at random using sampling with replacement. On the other hand, we tried making up for the differences in scores in the left and in the right hemisphere by training not only on our original training set, but also on the same training set flipped vertically. In this way, the left hippocampus was found in the spot of the right hippocampus and vice-versa. The criteria were adjusted accordingly.

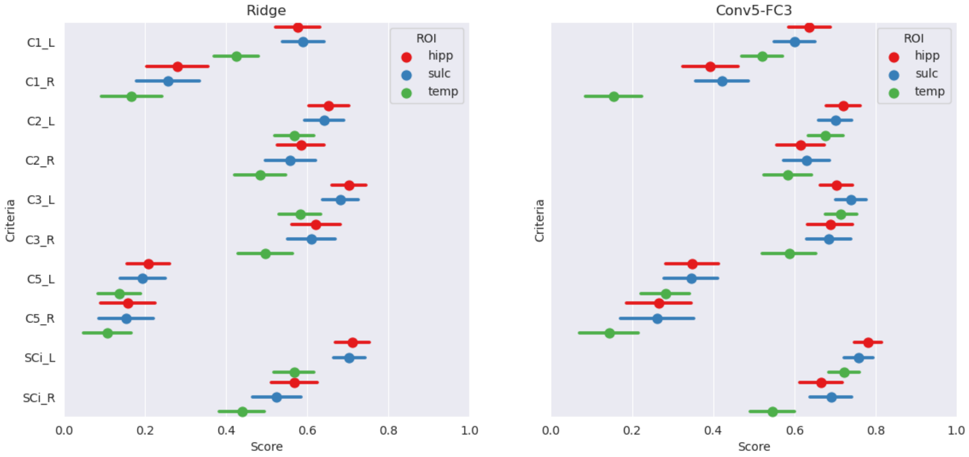

Figure A5: *Perfomances using three ROIs.* We show the mean metrics (weighted kappas for C1 C2 and C3, an unweighted kappa for C5 and ICCs for SCi) and 95% confidence intervals obtained through bootstraping. Results are shown for the ridge regression (left) and the Conv5-FC3 or CNN (right) for three ROIs: the hippocampus and close sulci (hipp), the hippocampus and all surrounding sulci (sulc) and the entire temporal lobe (temp).

A.2.1 Results

ROI: The effect of the ROI choice on the performance is presented on Figure A5. The smallest ROI achieved at least similar (if not better) performance on all criteria as larger ROI. As it is computationally more efficient to use a smaller ROI, we perform subsequent tasks using this ROI.

Data augmentation: We examine the performances of composite score predictions with over-sampling, with the addition of a flipped data-set and without data-augmentation on a pooled test set of all cohorts (N=502+248+100+246), comparing the results of the single training strategy (IMAGEN) and MUTLI (IMAGEN,QTIM,QTAB). Results are displayed in Figure A4 for the Conv-FC3 model. Human performances (inter and intra-rater ICCs) are plotted for reference.

None of our attempts at improving the training showed a significant improvement over the conv5-FC3 model trained on IMAGEN, QTIM and QTAB, in the right or left hemisphere.
